# A New Classification of the Morphology of Complete Ponticulus Posticus on Cone Beam Computed Tomography

**DOI:** 10.3390/diagnostics13183009

**Published:** 2023-09-21

**Authors:** Raphael Olszewski, Julien Issa, Guillaume-Anthony Odri

**Affiliations:** 1 Department of Oral and Maxillofacial Surgery, Cliniques Universitaires Saint Luc, UCLouvain, Av. Hippocrate 10, 1200 Brussels, Belgium; 2 Oral and Maxillofacial Surgery Research Lab (OMFS Lab), NMSK, Institut de Recherche Expérimentale et Clinique, UCLouvain, 1200 Brussels, Belgium; 3 Department of Diagnostics, Poznań University of Medical Sciences, Bukowska 70, 60-812 Poznań, Poland; julien.issa@student.ump.edu.pl; 4 Doctoral School, Poznań University of Medical Sciences, Bukowska 70, 60-812 Poznań, Poland; 5 DMU Locomotion, Service de Chirurgie Orthopédique et Traumatologique, AP-HP, Hôpital Lariboisière, Université Paris Cité, 75013 Paris, France; gaodri@gmail.com; 6 BIOSCAR UMRS 1132, INSERM, 75010 Paris, France

**Keywords:** ponticulus posticus, CBCT, cone beam computed tomography, atlas, cervical vertebra

## Abstract

The objectives of this retrospective study were to measure the prevalence of complete ponticulus posticus (CPP), to propose a new classification based on two different shapes of CPP, to compare these shapes with age and gender, and to test two different methods of measurements of the diameters of CPP on cone beam computed tomography (CBCT). Material and methods: We used 2012 CBCT scans from Planmeca Promax 3D Mid and Romexis 5.1 software tools to measure the height and width of the CPP, and we measured the surface of the CPP using an ellipse tool. We classified the CPP into “thin” and “thick” shape. Results: the prevalence of CPP was 9.49% with 97 male and 94 female patients. The unilateral type was found in 131 patients, while the bilateral type was found in 60 patients. Intra-observer reliability was evaluated using the intraclass correlation coefficient (ICC). The ICC was 0.875 for height, 0.872 for width, and 0.885 for the ellipse area. Both methods present very good intra-observer reproducibility. The “thin” group tended to be older and significantly more related to female patients. The “thick” group was associated with younger male patients. Conclusions: the proposed classification of CPP may be used when reporting the CBCT large field of view. There is still a need to increase the knowledge on the atlas and on its main variant, such as complete PP.

## 1. Introduction

Ponticulus posticus (PP) is an anatomic variant of the first cervical vertebra (atlas) and was first described by Bolk in 1906 [1]. PP is a partial or complete bone bridge situated between the posterior portion of the superior articular process of the atlas and the posterior and lateral portion of the superior edge of the posterior arch of the atlas [2,3]. 

PP also has multiple other names in the medical literature, such as dorsal or posterior ponticle [1], posterior ponticulus [4], pons posticus [1], pons ponticus [4], sagittal foramen [1,5,6,7], atlantal posterior foramen [5], foramen atlantideum [1,8], foramen atlantoideum posterior [6,7], posterior atlantoid foramen, [4], arcuate foramen [1,4,5,7,8], arcuale foramen [1,6,7], atlas bridging [4,8], a variant of Kimmerle’s anomaly [1,5,6,7], upper retroarticular foramen, [5], retroarticular canal [5,6], retroarticular foramen [4,8], foramen retroarticulare superior [6], retroarticular canal of the atlas [1], canalis vertebralis [1,5,6,7,8], canalis arteriae vertebralis [4], retroarticular vertebral artery ring [1,4,5,6], retrocondylar vertebral artery ring [5,6,8].

PP represents the ossification at the edge of the lateral atlantooccipital membrane, which is inserted on the occipital bone and on the edge of the posterior arch of the atlas [9,10]. Many hypotheses exist to explain the ossification of this membrane, such as congenital [4,6,11], genetic mutation [4,6,12], post-traumatic genesis [12], human evolution [12] with a protective role to the vertebral artery passage during head and neck movements [4], the result of ossification due to aging [4,6], and due to external mechanical factors [6] such as carrying heavy objects on the head [4].

PP surrounds the vertebral artery, periarterial plexus [5], and the suboccipital nerve [4]. The compression of one or more neural and vascular structures, including vertebral artery, periarterial plexus, and sub-occipital nerve passing under the PP [3,4,10], may explain the presence of diverse symptoms including muscle tension headache [4,6,12], cervical migraine [3,5,10,13], migraine without aura [8], migraine-like headache [2], cochlear symptoms (tinnitus and hearing loss) [12], neurosensory-type hearing loss [3,4,5,6,10], vestibular symptoms (subjective vertigo [2,3,5,6,8,10,12]), dizziness [4], ocular symptoms (convergence deficit) [12], diplopia [8], throat disorders (dysphagia, dysphonia) [12], cervicobrachial syndrome [12], neck pain [2,3,4,5,6,8,10], shoulder–arm pain [3,4,5,6], loss of postural muscle tone [3,4,5,10], loss of consciousness [3,4,5,10], syncopal crisis, [12], vertebrobasilar insufficiency syndrome [3,4,5,6,10], vertebral artery compression [6] or vertebral artery dissection [6], and Barré–Liéou syndrome [4]. Headache, neck, and shoulder/arm pain, as well as vertigo, have been found with significantly greater frequency in patients with complete PP compared with partial PP [3,5]. However, most of the patients with PP are symptom-free [6].

Furthermore, PP is an important region for the treatment of atlantoaxial instability [14]. Atlantoaxial instability is characterized by excessive movement between the atlas and the axis, potentially giving rise to a range of symptoms like balance problems, blurred vision, vertigo, and headaches [14]. The main treatment of the atlantoaxial instability (excessive movement at the junction between the atlas and the axis) [7] consists of the lateral mass screw fixation for the stabilization of the atlas [5,6,14,15] through the posterior arch [7,16]. Damage to the vertebral artery with screw fixation may appear if PP is overlooked or mistaken for a thick posterior arch of C1 [8,10]. The injury of the vertebral artery with the screw may provoke artery hemorrhage [2], stroke, or death by thrombosis, embolism, or arterial dissection [8]. 

Currently, there are various classifications of PP in the literature: complete/partial [8,13], unilateral/bilateral [5], a three-step scale (involving less than 50% of the arch length, more than 50% of the arch length, and complete PP) [15], and the four-step scale classification (small incomplete PP, less than 50% around the artery, more than 50% around the artery, and complete PP) [6,12,17]. The prevalence of PP varies across different studies and populations, ranging from 1.3% to 45.9% [2]. Most previous investigations were performed on dry skulls [10,18], lateral X-rays, and CT scans [19]. More recent studies have implemented cone beam computed tomography (CBCT) [19]. Nonetheless, the application of two-dimensional (2D) technologies (lateral X-rays) falls short in accurately assessing the morphological attributes and symmetry anomalies of PP [4]. Presently, CBCT is recognized as the gold standard in the radiodiagnosis of the PP, facilitating three-dimensional (3D) classification [4,15]. 

The objectives of this single-centered retrospective study were to assess the prevalence of PP in a cosmopolitan European capital city, to propose a novel classification system based on two distinct shapes of complete PP, and to analyze their correlation with age and gender. Furthermore, the study aimed to evaluate the effectiveness of two different measurement methods for determining the diameters of complete PP on CBCT. 

## 2. Materials and Methods

This retrospective study obtained the ethical approval from the Ethical Committee of Cliniques Universitaires saint Luc (UCLouvain) (B403/2019/03DEC/542). All CBCT scans were performed for other reasons than those for this study.

### 2.1. Searching Strategy

For the introduction, results, and discussion section, we choose articles in a reproducible manner using search queries from two databases: PubMed and Google Scholar. The search was conducted by a single observer. The focus was on articles written in English. The inclusion criteria encompassed studies about complete PP when using CBCT: clinical studies, case series, case reports, studies on living patients, and studies including information on complete PP. 

Conversely, the exclusion criteria were studies involving dry skull/vertebrae, studies on PP using other medical imaging (CT scan, lateral X-ray), studies beyond the intended scope, animal studies, forensic-related studies, and studies lacking comprehensive information on the specific type of PP.

The search query for PubMed was set as follows with mesh terms “ponticulus posticus” and “CBCT”: (“cervical atlas” [MeSH Terms] OR (“cervical” [All Fields] AND “atlas” [All Fields]) OR “cervical atlas” [All Fields] OR (“ponticulus” [All Fields] AND “posticus” [All Fields]) OR “ponticulus posticus” [All Fields]) AND “CBCT” [All Fields]. The search was performed on 16 December 2022, and 18 articles were collected. 

The search query for Google Scholar was set as follows: “Ponticulus posticus CBCT.” The search was performed on 9 January 2023. Initially, 169 articles were obtained. After the application of inclusion and exclusion criteria, only 15 articles were included in our review [2,3,4,5,6,7,8,10,11,12,13,14,15,16,17]. 

We cross-checked the finally retained articles from Google Scholar with PubMed. In 18 articles from PubMed, there were 10 duplicates from Google Scholar, and 7 articles without information about complete PP. Only one article from PubMed was finally retained for our review, but it was rejected after full paper reading (out of scope).

### 2.2. Measurement Methodology

We revised 2228 consecutive CBCT scans retrieved from Cliniques universitaires saint Luc (UCLouvain). We excluded 216 CBCT scans where the atlas (C1 vertebra) was not fully visible. Finally, we retained 2012 CBCT scans. No information was provided about the patient’s medical history or symptoms of disease in the head and neck area [11]. 

**Figure 1:**
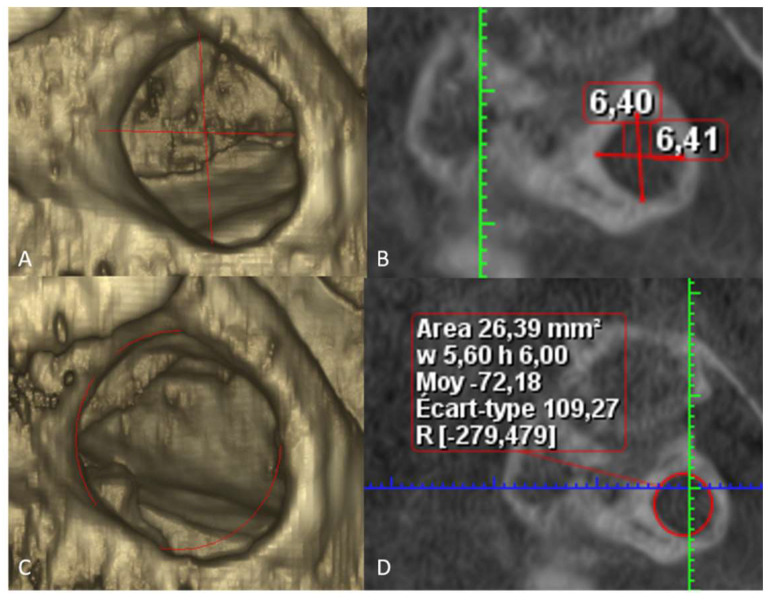
Methodology of measurements of complete PP. (**A**) 3D reconstruction of the complete PP, with visualization of the height and length (red lines). (**B**) Sagittal view, with automatic measurement of the height and width (red lines) in the region of the smallest diameters. (**C**) 3D reconstruction of the complete PP, with visualization of the area measurement with ellipse tool from Romexis software tool. (**D**) B. Sagittal view, with automatic measurement of the area of complete PP in the region of the smallest area.

Measurements were performed by one observer twice with one-month interval of time between measurements. We used CBCT Planmeca Promax 3D Mid (Planmeca, Helsinki, Finland) with the following parameters: 90 kVp, 5.6 to 14 mAs, slices of 200 microns, and diverse fields of view: 16 × 6.2 cm, 16 × 10.2 cm, and 20 × 17.4 cm. Planmeca Romexis 5.1 software tools were used for the measurements. We worked only on 2D sagittal views where PP is best visible in clinical practice [14]. We performed two sets of measurements: height and width of the complete PP, and the surface of the PP using ellipse tool. The measurements were provided by the software in mm for diameters and in mm^2^ for the area of PP (Figure 1). All the measurements were performed in the region of the smallest diameters and areas of the complete PP.

### 2.3. Classification Definition

**Figure 2:**
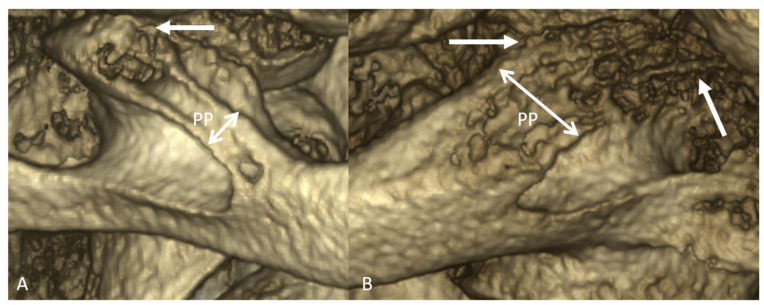
Classification of complete PP. (**A**) Complete PP with “thin” type. Arrow shows the insertion of PP on the lateral portion of the superior and posterior edge of the articular eminence. (**B**) Complete PP with “thick” type. Arrows show the insertion of PP on all the length of the superior and posterior edges of the articular eminence.

We were provided with original and new definition of complete “thin” PP and complete “thick” PP. The thin complete PP represents the bone bridge linking the lateral portion of the superior and posterior edge of the articular eminence and the posterior arch. The thick complete PP represents the bone canal linking all the length of the superior and posterior edge of the articular eminence and the posterior arch (Figure 2).

### 2.4. Statistics Methods 

Data for this study were handled in Microsoft Excel prior to analysis. Intra-observer reliability was evaluated using the intraclass correlation coefficient (ICC) two-way random single measures absolute agreement model (2, 1) in IBM SPSS Statistics (version 21.0.0.0). ICC values between 0.75 and 1.00 were considered exceptional. For continuous variables, means and standard deviations were calculated between groups and compared using either ANOVA or Student’s *t*-test as appropriate, with JMP^®^ 7.0.1 (SAS Institute Inc., Cary, NC, USA) software. Chi-squared test was used to compare categorical variable distributions between groups.

## 3. Results

### 3.1. Descriptive Statistics

In our study, 251 instances of complete PP were found in 191 patients, and the prevalence of complete PP was (191/2012) × 100 = 9.49%. There were 97 male and 94 female patients. The mean age was 47.3 ± 21.4 years old (range between 25.9 and 68.7 years old). The unilateral type was found in 131 patients, while the bilateral type was found in 60 patients.

### 3.2. Analytic Statistics

Our first question was to know which of the two methods of measurement of internal dimensions of complete PP was the most accurate: the two main dimensions technique (height × width) compared to the ellipse area technique. The ICC for the height was 0.875, the ICC for the width was 0.872, and the ICC for the ellipse area was 0.885. Notably, both methods exhibited excellent intra-observer reproducibility. 

Our second question was to ascertain whether a significant distinction exists between the “thin” and “thick” groups (Table 1). Additionally, we explored potential significant differences between cases of unilateral and bilateral complete PP (Table 2). The “thin” group tended to be older and was significantly more related to female patients. The “thick” group was associated with younger male patients (Table 1). The morphology was significantly thicker in the bilateral group than in the unilateral group (Table 2). 

Our last question was to investigate potential correlations between the morphology categories (“thin”, “thick”) of complete PP and the measurements of PP obtained using both methods (two main dimensions technique (height × width) compared to the ellipse area technique) (Table 1). Additionally, we aimed to assess whether a relationship existed between unilateral or bilateral complete PP and the age of the patient (Table 2). 

The analysis did not reveal any significant differences between the measurements of PP and their corresponding morphologies (confirmed through ANOVA test and Student’s *t*-test). No significant difference was observed in relation to the association between unilateral or bilateral complete PP and patient age (*t*-test of Student, *p* = 0.56) (Table 2).

## 4. Discussion

The prevalence found in our study (9.49%) follows the prevalence from studies from USA [17], Italy [4], Turkey [2,3,5,7,10,14,15,16] (Turkey, mean 9.96%, with minimum 3.70% and maximum 16.23%), and from India [13] (Table 3). Complete PP seems to be a rarer anatomical variant in Taiwan and Japan [7] (Table 3). All selected studies [2,3,4,5,6,7,10,11,12,13,14,15,16,17] presented partial and comp. However, we were able to find the data relating to complete PP and calculate its prevalence in most of the articles (Table 3). There also exists an overrepresentation of data from Turkey, with half of the 16 included studies published from Turkey [2,3,5,7,10,14,15,16].

Male/female prevalence and the mean age were calculated for all the samples (including partial and complete PP) in all the selected studies [2,3,4,5,6,7,10,11,12,13,14,15,16,17] (Table 3). Therefore, we were not able to directly compare our findings, which are only based on complete PP. 

Sekerci et al. [5,10] and Bayrakdar et al. [7] found that the distribution of the presence of PP was higher in males than in females [5,7,10]. We found the same distribution in male and in female for the presence of complete PP (Table 1 and Table 2). 

Unilateral type was more frequently present in our study, aligning with the findings of with Chen et al. [11], Buyuk et al. [16], Sabir et al. [13], Sekerci et al. [5], Hasani et al. [8], and Bayrakdar et al. [7] (Table 3). We also found that the morphology was significantly thicker in the bilateral group than in the unilateral group.

Macri et al. [4], Sekerci et al. [5], Hasani et al. [8], Chen et al. [11], and Tripodi et al. [12] found no association between chronological age and presence of the PP and between chronological age and types of the complete PP (unilateral, bilateral) [4]. Hasani et al. [8] found that 20–34 years and <45 years age groups demonstrated a higher prevalence of PP (partial and complete) than other groups. In contrast, we found that there exists a tendency between the chronological age and the proposed new types of complete PP (“thin”, “thick”).

Macri et al. [4] concluded that a progressive ossification of the PP with increasing age is the cause of bilateral complete variant increase with a concomitant bilateral partial reduction. We examined an intermediate population (25.9 to 68.7 years old), as compared to Macri et al. [4] (6 to 87 years old), and searched only for complete PP morphology. To demonstrate the presence of a progressive calcification of PP and transformation from partial PP to complete PP in the same group of patients, it is imperative to track and observe this cohort over an extended duration. However, we found that the “thin” morphology of complete PP (bone bridge) tended to be more associated with older patients, and the “thick” morphology of complete PP (canal shaped) was associated with younger patients, which is against the hypothesis of Macri et al. [4]. Moreover, Tripodi et al. [12] found cases of bilateral complete and unilateral complete PP before the age of 10 years. Geist et al. found 60 complete PP in a cohort of 576 patients from 10 to 17 years old and proposed a congenital or genetic origin of calcification of PP [17]. It is also possible that no single explanation exists of the origin of complete PP, and many explanations of the presence of complete PP may co-exist.

Hasani et al. found that the mean height of PP was 5.95 mm (SD 0.7823), and the mean width was 6.52 mm (SD 1.0308) [8]. Michell et al. stated that the super inferior diameter of PP was significantly less than the anteroposterior diameter [20]. We found that the super inferior diameter of complete PP was less important than the anteroposterior diameter on the sagittal view. Moreover, we tested the technique of ellipse to measure the area of complete PP, and it showed very good intra-observer reproducibility. The ellipse technique may be used as an alternative to the height/width measurement in deformed complete PP, where the definition of vertical and horizontal diameter may differ for different observers. 

The “thin group” was significantly more related to female patients. This additional finding may be of interest in forensic medicine to help in establishing the gender of a victim’s remains [21]. 

The main study limitation is related to the absence of a correlation of our findings with potential clinical symptoms associated with the presence of thin or thick complete PP [5,6]. We were not able to measure the thickness of the complete PP on the 3D CBCT reconstruction due to a lack of 3D tools in the Romexis Planmeca software tool. 

Finally, the cervical vertebrae are always inside the field of view when selecting large fields of view on CBCT. Extragnathic areas, such as the cervical vertebra, are less known by dentists and maxillofacial surgeons because they are out of their main clinical scope. However, CBCT users have an obligation to interpret all the selected fields of view [22,23,24], and if using a large field of view, they also need to increase their knowledge of the cervical vertebra and its main variant such as complete PP. 

## Figures and Tables

**Table 1:** Comparison between thin and thick morphology of complete PP. F, female; M, male; L, left; R, right.

	Thin	Thick	*p*-Value
Age in years (mean ± SD)	50.5 ± 21.6	45.4 ± 21.2	0.0633
Gender (F/M)	63/46	57/85	0.0054 *
Side (L/R)	58/51	72/70	0.07
Hight in mm (mean ± SD)	6.7 ± 1.1	6.5 ± 1.0	0.15
Width in mm (mean ± SD)	7.5 ± 1.1	7.3 ± 0.9	0.24
Area in mm^2^	32.8 ± 7.9	31.5 ± 7.1	0.18

* Significance level (*p*-value) ≤ 0.05.

**Table 2:** Comparison between unilateral and bilateral groups. F, female; M, male.

	Unilateral	Bilateral	*p*-Value
Age in years (mean ± SD)	47.1 ± 21.2	47.8 ± 22.0	0.83
Gender (F/M)	68/63	26/35	0.23
Morphology (Thin/Thick)	68/63	41/79	0.0045 *
Hight in mm (mean ± SD)	6.5 ± 1.1	6.7 ± 1.0	0.17
Width in mm (mean ± SD)	7.4 ± 1.0	7.4 ± 1.0	0.72
Area in mm^2^	32.0 ± 7.9	32.1 ± 7.0	0.84

* Significance level (*p*-value) ≤ 0.05.

**Table 3:** Overview of studies reporting complete PP. * CLP: cleft lip palate.

Author, Year of Publication	Country	CBCT Scans	Number of Complete PP in the Study	Prevalence (%) of Complete PP	Unilateral/Bilateral
Geist et al., 2014 [17]	USA	576	60	10.41	No information
Bayrakdar et al., 2014 [15]	Turkey	730	70	9.58	48/79
Sabir et al., 2014 [13]	India	200	17	8.5	13/4
Chen et al., 2015 [11]	Taiwan	500	23	4.60	20/3
Sekerci et al., 2015 [5]	Turkey	698	112	16.04	68/44
Sekerci et al., 2015 [10]	Turkey	542	70	12.91	34/36
Hasani et al., 2016 [8]	Iran	260	21	8.07	15/6
Buyuk et al., 2017 [16]	Turkey	374	40	10.69	21/19
Bayrakdar et al., 2017 [7]	Turkey	181	16	8.83	9/7
Joshi et al., 2018 [6]	Japan	30	No information	No information	10/unknown
Bayrakdar et al., 2018 [14]	Turkey	54 (CLP) *, 108 (control group)	4 (CLP), 4 (control group)	7.40 (CLP), 3.70 (control group)	2/2 CLP, 2/2 control group
Tripodi et at., 2019 [12]	Italy	524	24	4.58	7/17
Evigren et al., 2020 [2]	Turkey	440	55 (left), 63 (right)	12.5 (left), 14.30 (right)	118/unknown
Macri et al., 2021 [4]	Italy	500	42	8.4	18/24
Polat et al., 2022 [3]	Turkey	171	15	8.77	No information

## Data Availability

The data that support the findings of this study are available from the corresponding author upon reasonable request.
